# Effect of laser fluence on the optoelectronic properties of nanostructured GaN/porous silicon prepared by pulsed laser deposition

**DOI:** 10.1038/s41598-023-47955-3

**Published:** 2023-11-29

**Authors:** Makram A. Fakhri, Haneen D. Jabbar, Mohammed Jalal AbdulRazzaq, Evan T. Salim, Ahmad S. Azzahrani, Raed Khalid Ibrahim, Raid A. Ismail

**Affiliations:** 1 Laser and Optoelectronic Department, University of Technology-Iraq, Baghdad, Iraq; 2 Applied Science Department, University of Technology-Iraq, Baghdad, Iraq; 3 Electrical Engineering Department, Northern Border University, Arar, Kingdom of Saudi Arabia; 4 AlFarahidi University, Baghdad, Iraq

**Keywords:** Materials science, Nanoscience and technology, Optics and photonics

## Abstract

In this study, the fabrication of nanostructured GaN/porous Si by pulsed laser deposition (PLD) was demonstrated. The porous silicon was prepared using laser-assisted electrochemical etching (LAECE). The structural, optical, and electrical properties of GaN films were investigated as a function of laser fluence. XRD studies revealed that the GaN films deposited on porous silicon were nanocrystalline, exhibiting a hexagonal wurtzite structure along the (100) plane. Spectroscopic property results revealed that the photoluminescence PL emission peaks of the gallium nitride over porous silicon (GaN/PSi) sample prepared at 795 mJ/mm^2^ were centered at 260 nm and 624 nm. According to topographical and morphological analyses, the deposited film consisted of spherical grains with an average diameter of 178.8 nm and a surface roughness of 50.61 nm. The surface of the prepared films exhibited a cauliflower-like morphology. The main figures of merit of the nanostructured GaN/P-Si photodetectors were studied in the spectral range of 350–850 nm. The responsivity, detectivity, and external quantum efficiency of the photodetector at 575 nm under − 3 V were 19.86 A/W, 8.9 × 10^12^ Jones, and 50.89%, respectively. Furthermore, the photodetector prepared at a laser fluence of 795 mJ/mm^2^ demonstrates a switching characteristic, where the rise time and fall time are measured to be 363 and 711 μs, respectively.

## Introduction

The category of semiconductor materials known as III-nitrides has gained popularity in recent years due to their wide and direct band gaps, as well as their capacity to create alloys like InGaN and AlGaN^1–3^. By adjusting the composition of these alloys, the band gap can be modified across the entire solar spectrum, from deep UV to IR^4–7^. GaN (gallium nitride), in particular, possesses a broad band gap of 3.4 eV and a wurtzite hexagonal structure, which results in minimal leakage currents and enables the operation of optoelectronic devices at elevated temperatures and frequencies^8–11^. GaN proves advantageous for optoelectronic applications, such as photodiodes, which find utility in diverse detection, monitoring, and control scenarios^12–15^. Furthermore, these photodiodes hold great promise for advanced uses, including military, medical, display, general illumination, and environmental monitoring applications^16–20^. Its distinctive characteristics also render GaN suitable for deployment in LEDs, solar cells, and photodetectors^21–24^.

Several techniques, including pulsed laser deposition, chemical vapor deposition, and molecular beam epitaxy, have demonstrated successful outcomes in producing GaN thin films. These methods share a common objective: the fabrication of high-performance P-N and P-I-N heterojunctions within GaN films of varying thicknesses and on diverse substrates. These aspects encompass efficiency, speed, responsivity, and minimal dark current^24–29^. Notably, the pulsed laser deposition method presents a straightforward protocol, generating a substantial, well-directed material plume^30–33^. Additionally, it offers meticulous control over growth rate and is well-suited for generating thin films with strong adhesion on cost-effective substrates. Furthermore, this technique enables precise regulation of thin film properties, encompassing thickness and structure^34–36^. n contemporary semiconductor manufacturing, silicon (Si) is extensively employed due to its cost-effectiveness and compatibility with various processes. However, silicon's applicability in the infrared spectrum is limited due to its heightened reflectance and wide band gap^37–39^. These constraints have been significantly alleviated with the advancement of porous silicon (P-Si) technology^40^. P-Si enhances surface area, rendering it a suitable substrate for optoelectronic devices^41^. Furthermore, porous silicon (P-Si) exhibits favorable characteristics like robust room-temperature photoluminescence (PL), elevated chemical reactivity, rapid oxidation, affordability, and a quantum confinement effect that enhances radiative transitions^42–46^. As a result, P-Si finds utility in the fabrication of various optoelectronic devices, encompassing photodiodes, LEDs, detectors, and even biosensors^47–49^. Deposition of a film on porous silicon for photodetection applications was reported^50^. This offers the advantages of a large sensitive surface area, the formation of two junctions connected in series, increased responsivity of the porous photodetector, and improved speed of response of the photodetector. Herein, a new device has been fabricated make use the advantages of two differnt teqniques photonic cysrtal substrate and nanofilm active layer. The fabrication of a high-performance GaN/PSi photodetector via the pulsed laser deposition method under various laser fluences has been reported.

## Experimental works

### Preparation of porous silicon substrates

**Figure 1:**
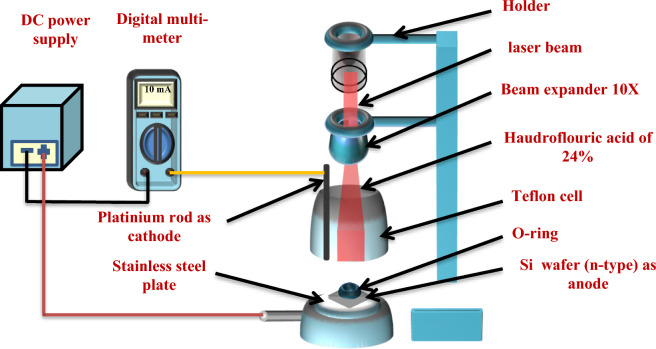
A schematic showing how a diode laser can aid the photo-electrochemical etching process^52^.

Mirror-like n-type (110) Si wafers with an electrical resistivity of 1–5 mΩ/cm and a thickness of 500 μm, which were purchased from University Wafer, Inc., USA, were utilized. Subsequently, the wafers were sectioned into rectangular pieces, each measuring 1 by 1 cm. Before initiating the photo-electrochemical etching process, the sections underwent a thorough cleaning using an ultrasonic device in ethanol (99.9% concentration, sourced from the German Honeywell company) for a duration of 5 min. The etching process was carried out at room temperature and involved the utilization of a diode laser (660 nm, 100 mW, from the Chinese Tongtool Company), a DC power supply with a voltage range of 0–30 V, and a digital multi-meter (Victor Company). This process, as depicted in Fig. 1, requires the use of a Teflon cell equipped with a cathode electrode made of 95% pure platinum and an anode electrode composed of silicon. The laser played a pivotal role in the top-down electrochemical etching technique employed for the synthesis of the porous silicon (PSi) substrates. Additionally, precise control was maintained over the etching conditions, with a designated etching time of 10 min, a consistently upheld current density of 10 mA/cm^2^, and a constant concentration of hydrofluoric acid (HF) (sourced from the German company Thomas Baker) at 24%, achieved through the use of the dilution equation^51^, as depicted in Eq. (1). The concentration of HF used in the etching process was consistently maintained at 24%, and the etching time was precisely set using a digital clock for a duration of 10 min^52–56^

1 $$ {\text{C}}_{1} {\text{V}}_{1} = {\text{C}}_{2} {\text{V}}_{2} $$

where C_1_, hydrofluoric acid concentration; V_1_, hydrofluoric acid volume; C_2_, ethanol concentration. V_2_, ethanol volume.

Upon completion of the LAECE process, all synthesized P-Si substrates undergo a series of tests to identify the optimal outcome for varying current densities^58–60^. Structural properties were examined using X-ray diffraction (XRD) equipment (XRD6000 Shimadzu Company) from Japan, utilizing copper radiation with a wavelength of 1.54060 Å. Morphological parameters were evaluated at a high level of detail using German field emission scanning electron microscopy (FESEM) technology (ZEISS Company). Surface characteristics were assessed using atomic force microscopy (AFM) equipment from the TT-2 Workshop Company in the United States. Spectroscopic features were analyzed using photoluminescence (PL) techniques from the Perkin Elmer Company in the United States of America.

### Preparation of gallium nitride pellet

A high-purity gallium nitride powder of 99.9%, purchased from Luoyang Advanced Material Company, was compressed into a pellet using a hydraulic compressor with a force of 10 tons. The GaN pellet was subjected to ablation using a Q-switching Nd:YAG laser (RY 280, China) with varying fluences ranging from 530 to 884 mJ/mm^2^. The laser had a wavelength of 355 nm and a pulse duration of 7 ns, and the ablation process was conducted under a vacuum pressure of 10^–2^ mbar. The deposition of the GaN film onto the PSi substrate occurred at room temperature. The structure of the GaN film deposited on PSi was examined using an X-ray diffractometer (XRD6000, Shimadzu Company). The morphology of the deposited films was studied using field emission scanning electron microscopy (FESEM) from ZEISS Company. The topography of the deposited films was investigated using an atomic force microscope. Furthermore, the photoluminescence (PL) properties of the films were analyzed using a spectrophotometer from Perkin Elmer.

### Electrical properties of GaN/PSi

**Figure 2:**
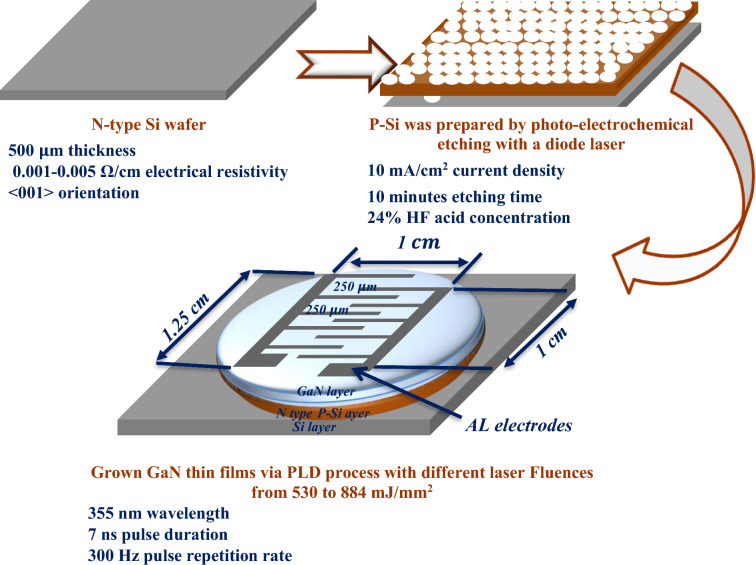
Process diagram of grown GaN nanostructure.

To measure the electrical properties of the GaN/PSi photodetector, a metal interdigitated mask was employed for establishing ohmic contacts. An aluminum film was deposited onto the GaN layer and the backside of the silicon substrate using the thermal evaporation technique, as depicted in Fig. 2. The current–voltage characteristics of the photodetector were measured at room temperature under both dark and illuminated conditions. This was achieved using a power supply (Dazheng 30 V, 5 A PS-305D from China) and digital multi-meters (UNI-T UT33C). Additionally, a programmable LCR meter (LCR-6100, Taiwan, GW Instek, 10 Hz–100 kHz) was employed to evaluate the capacitance–voltage characteristics of the photodetector.

### Figures of merit of the photodetector

The main figures of merit of the photodetector, namely responsivity (R), specific detectivity (D*), and external quantum efficiency (EQE) were measured using photodetector evaluation system. It is consists of monochromator (Jobin-Yuvon), beam spilter, halogen lamp, multimeter, and silicon power meter. These measurements were conducted out under a reverse bias of 3 V.

## Results and discussion

### XRD properties

**Figure 3:**
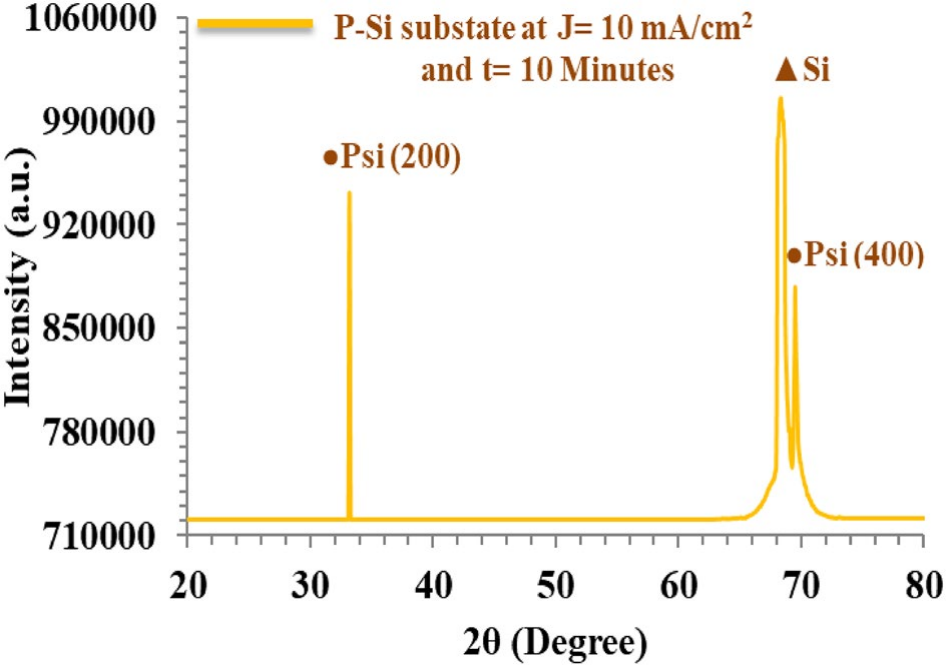
XRD pattern of PSi substrate.

Figure 3 depicts the XRD pattern of the PSi, revealing two peaks situated at 2θ = 33° and 68°, which correspond to the (200) and (400) planes, respectively. These two peaks are characteristic of porous silicon and align well with findings reported previously^61–66^. The XRD analysis of PSi confirms the splitting of the peak at 68° into two distinct peaks, representing crystalline silicon and porous silicon..

**Figure 4:**
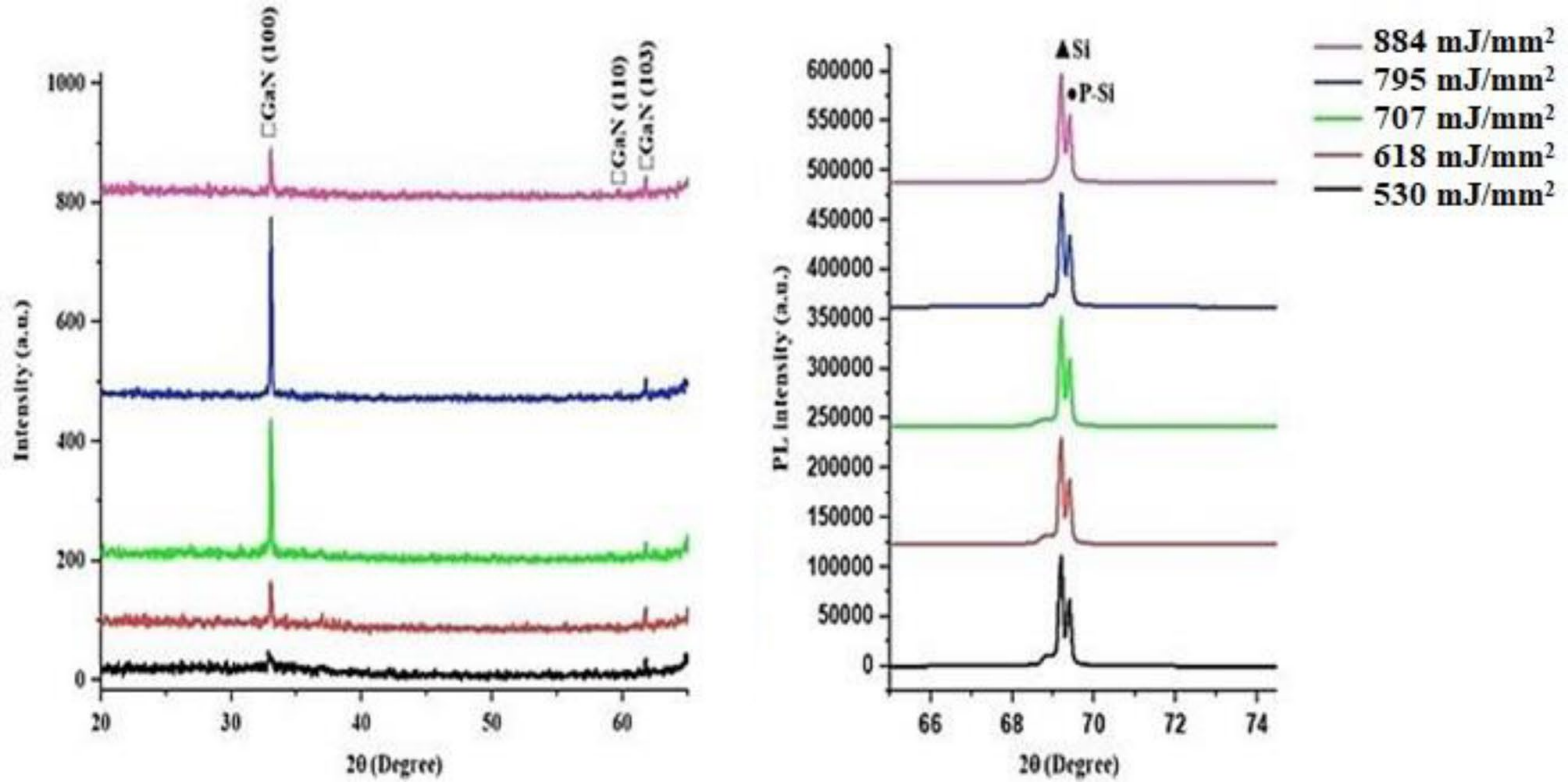
XRD pattern of GaN nano-crystalline films on a P-Si substrate using laser fluences ranging from 530 to 884 J.

Figure 4 depicts the XRD pattern of GaN nanocrystalline films deposited on PSi at various laser fluences. Three peaks were observed for all GaN films; these peaks are located at 2θ = 32.8°, 57.9°, and 61.7°, corresponding to (100), (110), and (103) planes, respectively. These peaks are indexed to GaN according to JCPDS # 01-074-0243. With an increase in laser fluence, a slight shift in 2θ was detected, and there was an observed increase in peak intensity along the (100) plane. The slight shift is attributed to stress and strain, while the increase in peak intensity is attributed to the greater film thickness and grain size.

The XRD analysis of the PSi substrate and the GaN films deposited on PSi is presented in Tables 1 and 2, respectively. To determine the crystallite size (D), Scherrer’s formula^67–70^ was employed, while the interplanar distance (d) was calculated using the formula^71–74^

2 $$ {\text{D}} = {{{\text K}\lambda }}/{\upbeta }\cos {{ \uptheta }} $$

3 $$ {\text{d}} = {{{\text n}\lambda }}/2{ }\sin {\uptheta } $$

where K is a constant set at 0.9, λ is the wavelength of the CuKα source, β is the fullwidth at half maximum of the XRD pattern,$\uptheta$ is the diffraction angle, and n is a positive integer.

**Table 1:** X-Ray diffraction pattern of fabricated P-Si layer.

Substrate orientation (hkl)	2 theta (Degree)	Full width at half maximum (Degree)	Crystallite size (nm)	Interplanner spacing (nm)
<200>	33.101	0.290	0.282	0.211
<400>	69.331	0.321	0.141	0.142

**Table 2:** XRD pattern of GaN nano-crystalline films over P-Si substrate.

Laser Fluence (mJ/mm^2^)	Film orientation (hkl)	2theta (Degree)	Full width at half maximum (Degree)	Crystallite size (nm)	Interplanner spacing (nm)
530	<100>	32.840	0.390	21.260	0.271
	<110>	57.921	0.170	53.471	0.152
	<103>	61.720	0.170	54.501	0.151
618	<100>	33.0	0.202	40.861	0.271
	<110>	58.921	0.150	60.900	0.152
	<103>	61.761	0.141	66.202	0.151
707	<100>	33.041	0.181	46.090	0.271
	<110>	59.160	0.230	39.762	0.152
	<103>	61.761	0.161	57.922	0.151
795	<100>	33.041	0.170	48.801	0.271
	<110>	59.801	0.061	152.932	0.152
	<103>	61.760	0.130	71.291	0.151
884	<100>	33.041	0.171	48.802	0.271
	<110>	59.761	0.050	183.481	0.152
	<103>	61.760	0.131	71.292	0.151

### Spectroscopic properties

**Figure 5:**
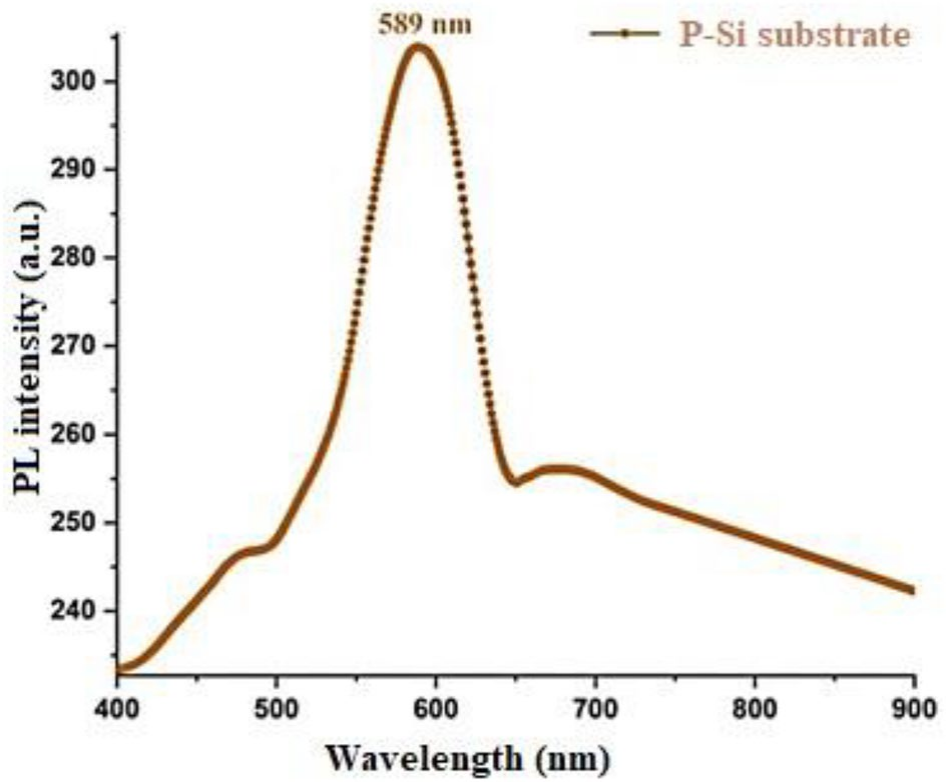
Room temperature photoluminescence prepared P-Si substrate.

The photoluminescence (PL) spectra of the PSi substrate are depicted in Fig. 5. PL measurements of the PSi substrate were conducted at room temperature with an excitation wavelength of 280 nm. Firstly, it is observed that the prepared PSi substrate exhibits an emission peak at 589 nm, which belongs to the visible yellow band. This peak is attributed to surface states and quantum confinement arising during the photo-electrochemical etching process, as mentioned by Wang^75–78^. The energy band gap of the prepared PSi substrate was determined to be 2.1 eV, larger than the energy band gap of crystalline silicon (1.11 eV). This difference in energy band gaps can be attributed to the combined effects of quantum confinement and increased surface states, altering the electronic structure of the material.

**Figure 6:**
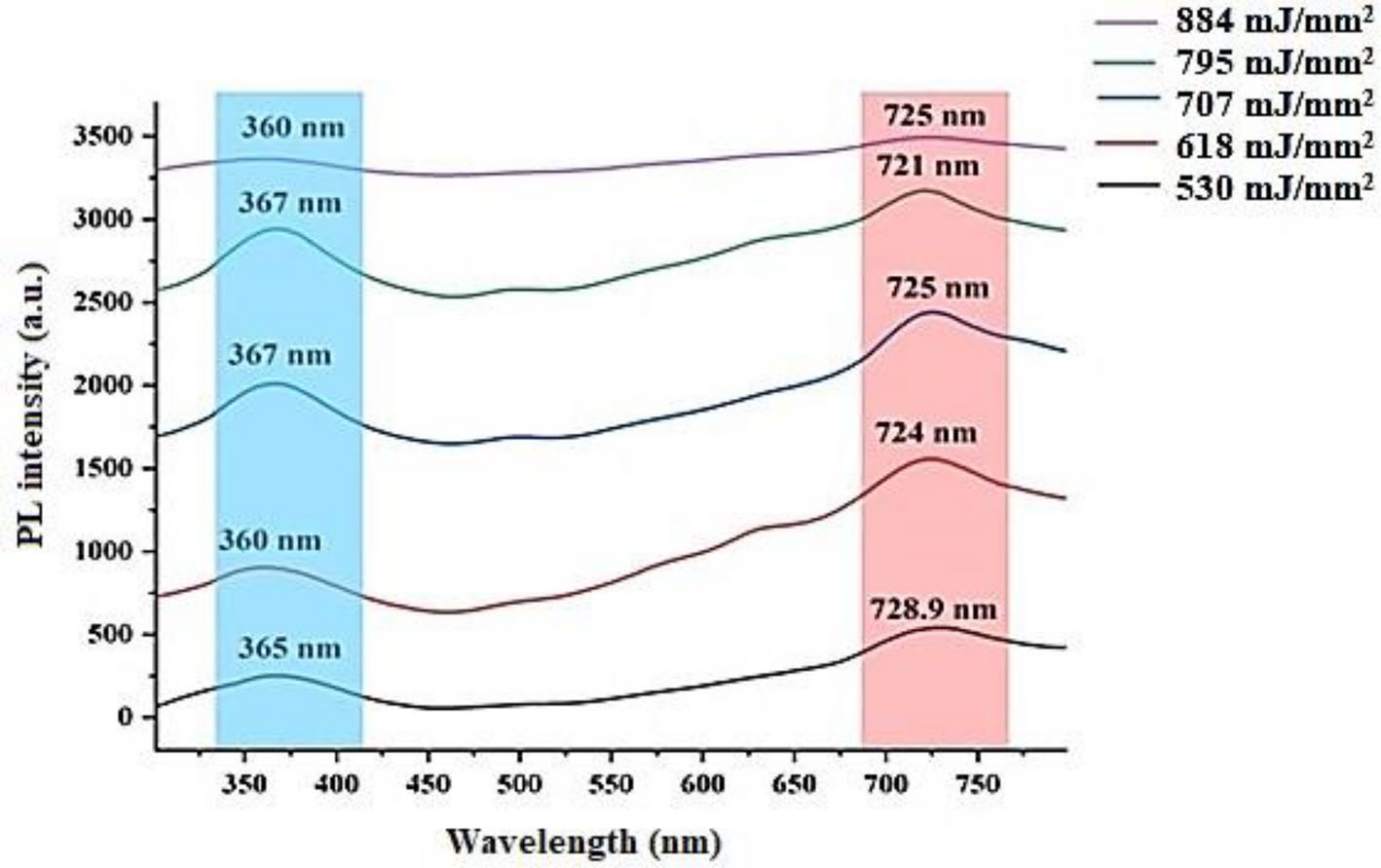
Room temperature PL spectra of GaN/PSi nanostructures prepared at various laser fluences.

The PL spectra of GaN nanocrystalline films prepared at different laser fluences are depicted in Fig. 6. The PL of GaN/P-Si nanocrystalline films was measured at room temperature, and the excitation wavelength was 320 nm. The PL spectra of the GaN nanocrystalline films exhibited UV bands located at 265.9, 267.9, 267, 260, and 260.9 nm, which are attributed to the GaN film, as well as red bands at 628.9, 621, 625.9, 624, and 625 nm, which belong to the P-Si substrate. Increasing the laser fluence led to a decrease in the PL intensity, but there were differing opinions regarding the peak location of the PL spectrum. This discrepancy was likely due to the higher defect density causing more non-radiative recombination. According to the PL results, the energy gaps for GaN nanofilms prepared at laser fluences of 530, 618, 707, 884, and 795 mJ/mm^2^ are 3.45, 3.38, 3.36, 3.34, and 3.44 eV, respectively, which are in agreement with the reported data^58,79–81^.

### Surface topography AFM

**Figure 7:**
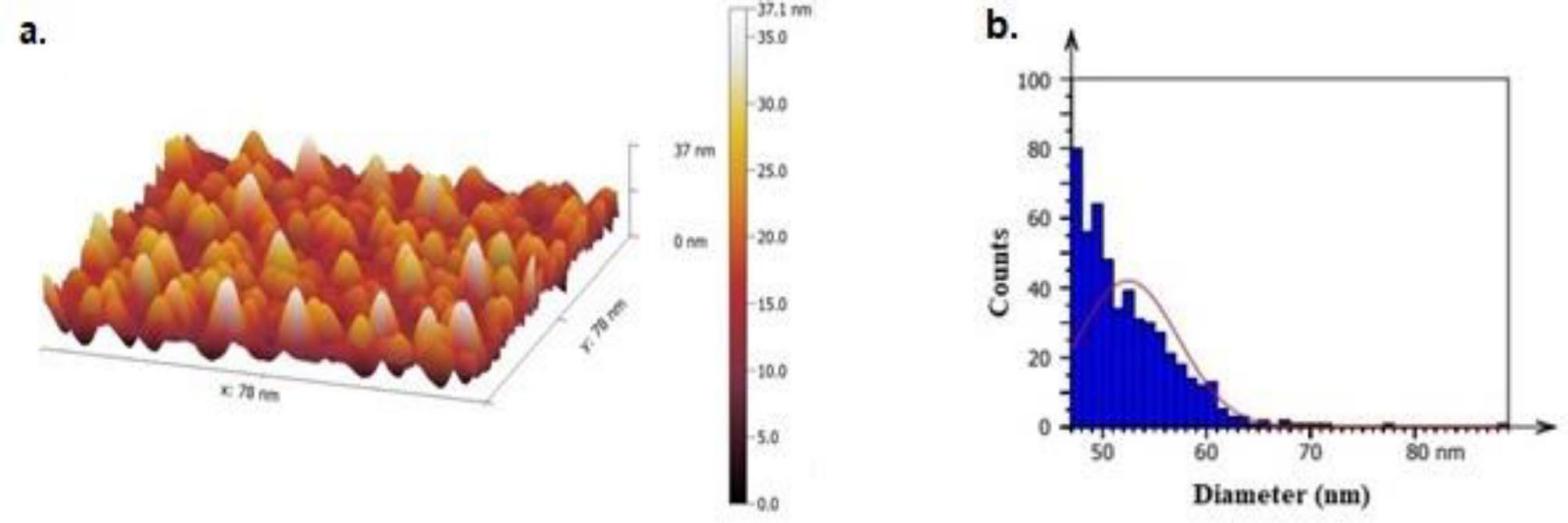
(**a**) AFM image of prepared P-Si substrate; (**b**) grain size distribution of prepared P-Si substrate.

Figure 7 a,b depicts the 3D AFM images and the grain size distribution of P-Si substrate etched. In contrast, after 10 min of etching, the pores formed uniformly throughout the entire surface, taking on a more elongated oval shape. Table 3 provides the AFM parameters of a prepared P-Si substrate. Most notably, nanometer-scale research into particle size distribution was conducted on the P-Si substrate after preparation.

**Table 3:** AFM parameters of PSi substrate.

Root-mean-square height (nm)	Maximum height (nm)	Average surface roughness (nm)	Average diameter (nm)
11.880	88.660	9.400	52.460

**Figure 8:**
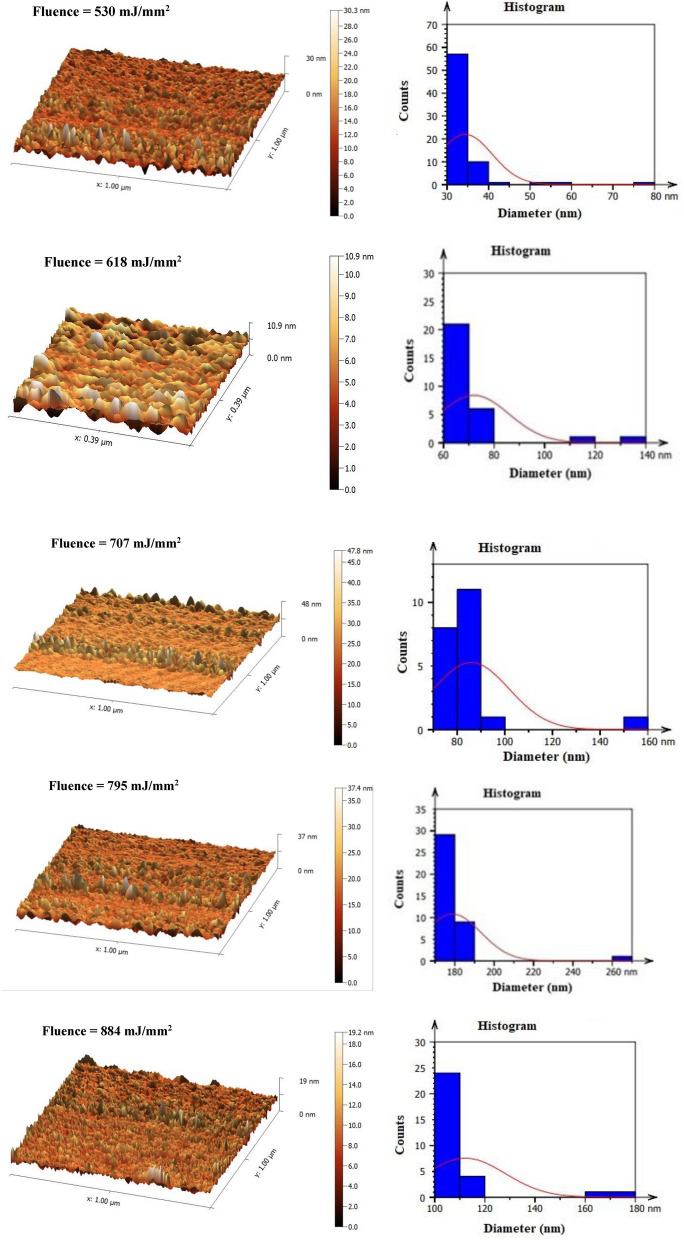
AFM and grain size distribution images of GaN/PSi nanostructures prepared at various laser fluences.

To analyze and characterize the topography of the prepared GaN nano-crystalline films over a PSi substrate, Fig. 8 depicts three-dimensional AFM images and grain size distribution. Average particle diameter and average surface roughness increased as laser Fluence increased from 530 to 884 mJ/mm^2^, but they decreased at 884 mJ/mm^2^, as demonstrated in Table 4. The AFM image of the GaN nanocrystalline film shows the uniform deposition of samples created with 795 mJ/mm^2^ laser Fluence and the largest average particle diameter and average surface roughness.

**Table 4:** AFM parameters of GaN/PSi nanostructures prepared at various laser fluences.

Laser fluence (mJ/mm^2^)	Root-mean-square height (nm)	Maximum height (nm)	Average surface roughness (nm)	Average diameter (nm)
530	15.962	78.011	13.832	34.411
618	34.411	131.701	28.423	72.051
707	46.822	154.321	42.521	85.932
795	59.012	265.301	50.610	178.83
884	33.853	179.702	26.012	112.222

### Surface morphology FESEM

**Figure 9:**
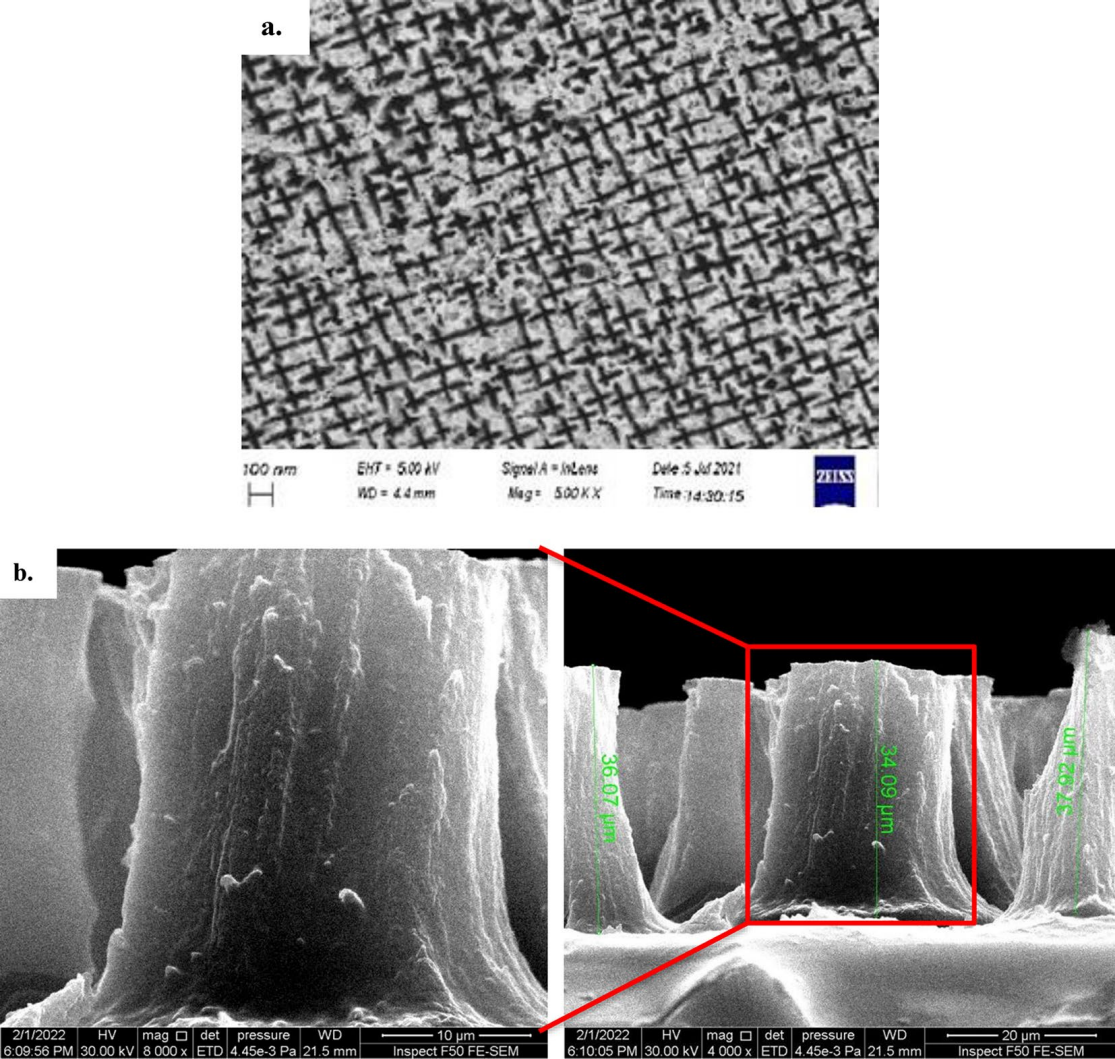
FE-SEM images of prepared P-Si substrate, a. surface area image, and b. cross section image.

Figure 9a and b depicts FE-SEM images of the surface and cross-section, respectively, of the PSi substrates, offering insights into the surface morphology. According to the research conducted by Omar et al.^67^, the pores on the surface exhibit a star-like appearance and maintain an elongated shape across the entire surface. This is attributed to the use of n-type silicon (100) with low resistivity during the preparation of the PSi^82–85^. Furthermore, the FE-SEM cross-sectional image revealed that the thickness of the P-Si layer measures 36.02 μm.

**Figure 10:**
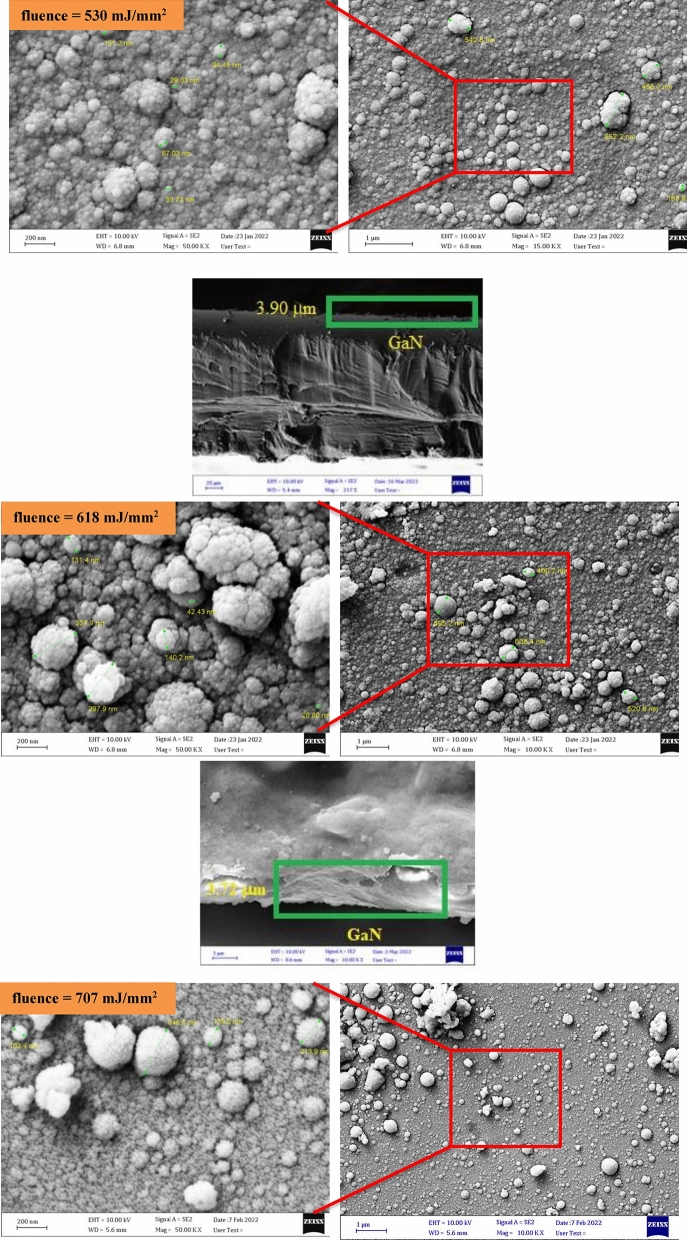

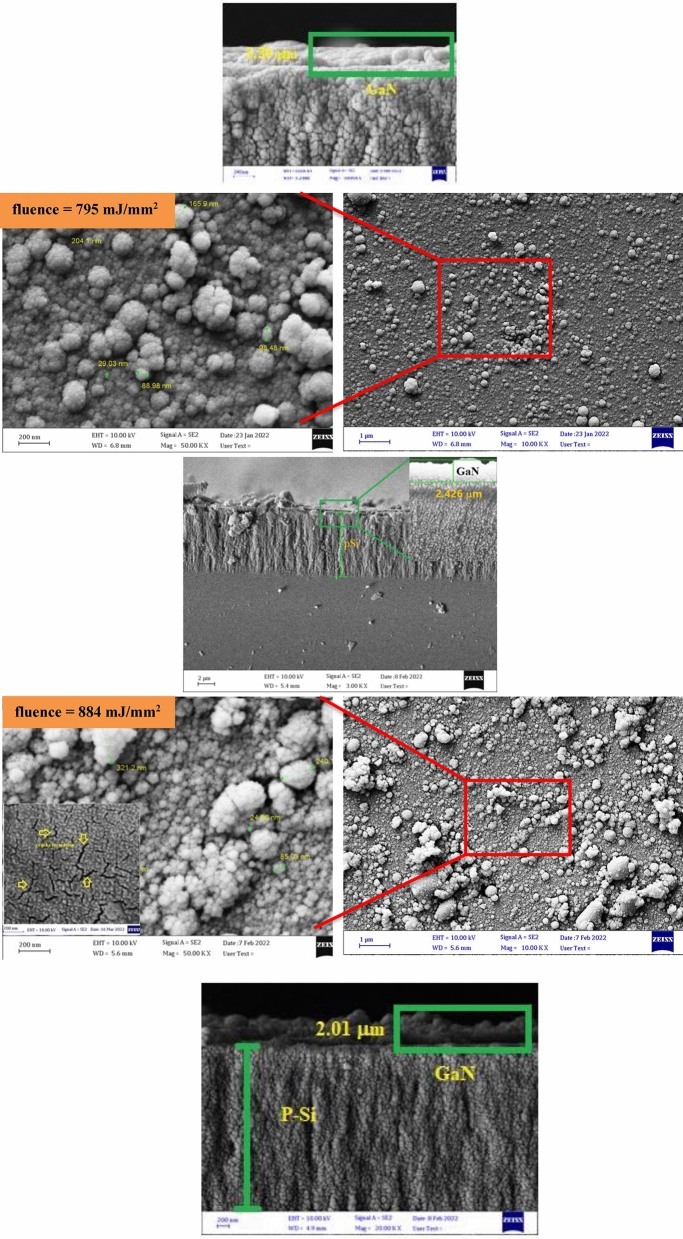
FE-SEM images and cross section images of GaN/PSi nanostructures prepared at various laser fluences.

**Figure 11:**
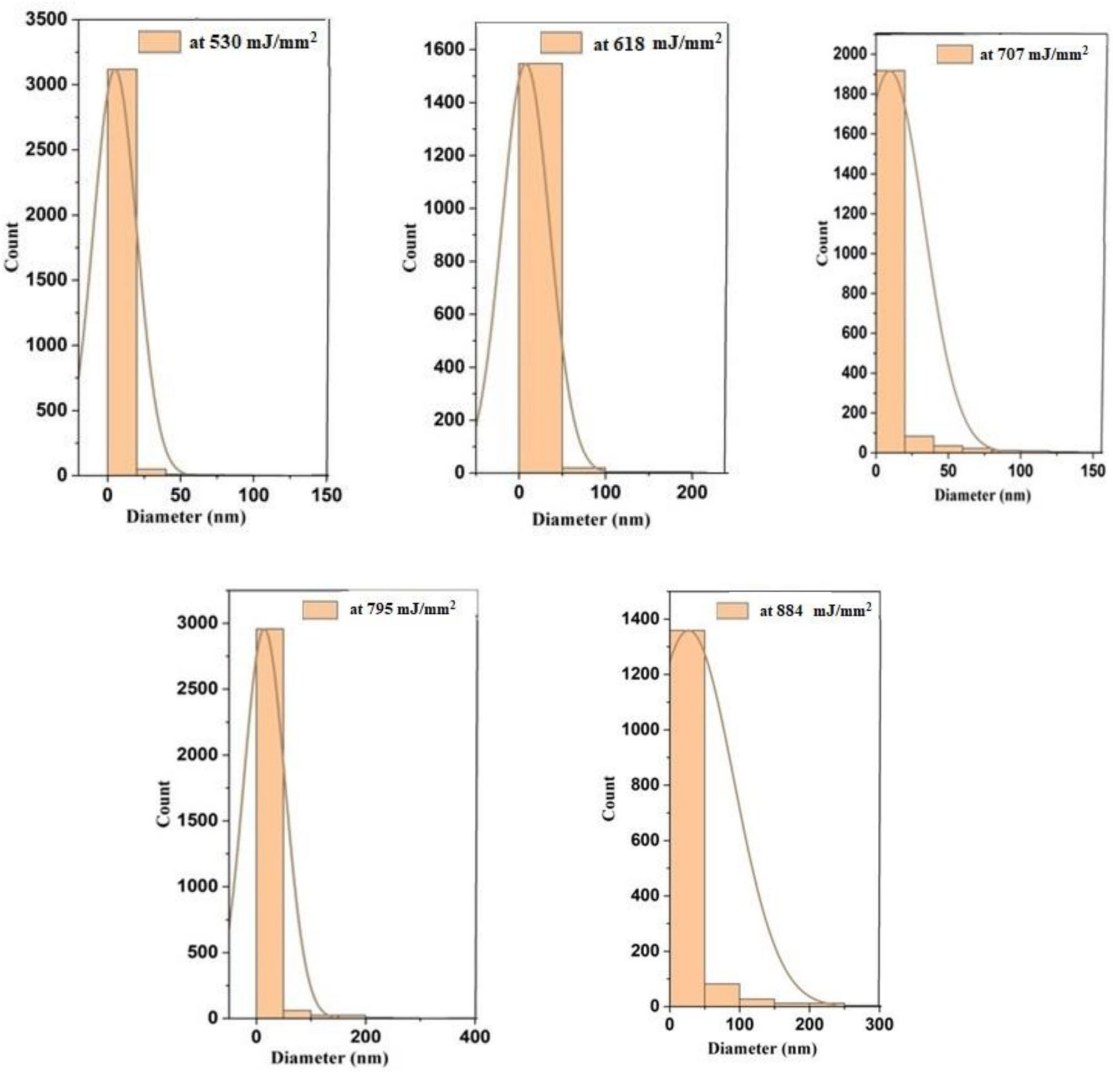
Average diameter images of GaN/PSi nanostructures prepared at various laser fluences.

Figure 10 depicts FE-SEM images of GaN/PSi nanostructures produced using PLD at varying laser intensities. This study revealed that the thickness of the GaN nanocrystalline film produced with a laser fluence of 795 mJ/mm^2^ during the PLD process measures approximately 383.36 nm, which is comparable to the thickness of the GaN nanostructures themselves. The surface morphology of the GaN films was analyzed using micro- and nano-scale techniques. As depicted in Fig. 11, the average diameter of GaN/PSi nanostructures created with different laser fluences was calculated through ImageJ analysis. Furthermore, the GaN nano-crystalline films fully covered the P-Si substrate, resulting in uniform and homogeneously-sized spherical particles with a cauliflower-like shape.

### Electrical properties

**Figure 12:**
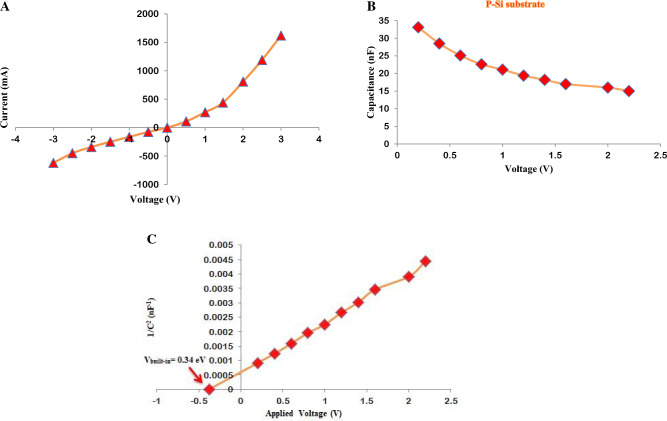
Electrical characteristic of prepared P-Si substrate, (**A**) dark I–V characteristic for forward and reverse bias, (**B**) C–V characteristic, (**C**) 1/C^2^ vs. voltage.

At room temperature, the dark current–voltage characteristic of the prepared P-Si substrate was analyzed in the dark, as depicted in Fig. 12A. As the voltage was applied, the current flowing through the P-Si substrate increased due to the elevated resistance of the P-Si layer with increasing voltage^86,87^. Moreover, the charge transfer led to the formation of a depletion zone in the prepared P-Si substrate close to the electrical dipole, resulting in a rectifying characteristic^88–90^.

Figure 12B depicts the capacitance–voltage characteristic for applied voltages ranging from 0 to 3 V. The capacitance of the prepared P-Si substrate decreased. This phenomenon has been coined as the “growing depletion region with increasing built-in potential”^57,91–94^.was coined to describe this phenomenon.

The relationship between 1/C^2^ and the voltage on the fabricated P-Si substrate is depicted in Fig. 12C. C^2^ exhibits a linear relationship with voltage. Figure 12C shows the correlation between 1/C^2^ and voltage on a prepared PSi substrate. a linear relationship with voltage. The built-in potential was determined by extending the given linear segment of the curve to a 1/C^2^ value of 0 points. There was an inherent potential of 0.34 eV.

**Figure 13:**
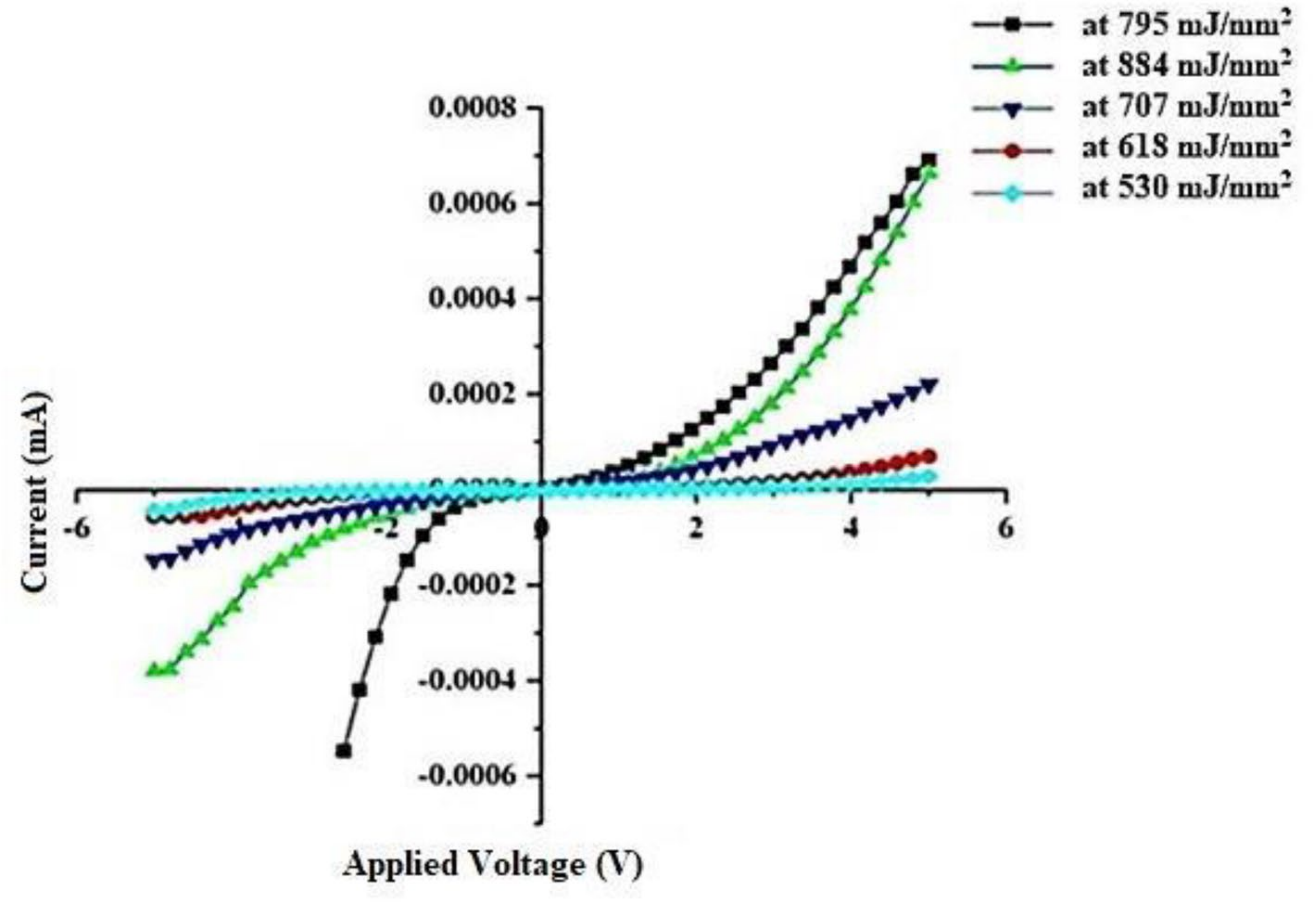
PLD-fabricated GaN/PSi nanostructure dark I–V properties at laser fluences between 530 and 884 mJ/mm^2^ under forward and reverse biases.

Figure 13 depicts the dark I–V characteristics of GaN nanocrystalline films fabricated on a P-Si substrate using the PLD method at various laser fluences and at room temperature. As the bias voltage was increased, the GaN nano-crystalline film created at 795 mJ/mm^2^ exhibited expansion due to the narrowing of the depletion layer^95,96^.

Furthermore, rectification features were observed in the GaN/P-Si nanocrystalline film, and recombination tunneling served as the current transport mechanism in both layers^97–99^.

### Performance characterization of GaN nanostructure with optimum laser fluence

The performance properties of the fabricated GaN/PSi heterojunction using the PLD method with optimal laser parameters (355 nm laser wavelength and 300 °C substrate temperature) at different laser fluences were determined, and they are illustrated in Figs. 15, 16, 17 and 18. The study concluded that a laser Fluence value of 795 mJ/mm^2^ was optimal. The responsivity (R_λ_), specific detectivity (D_λ_), and external quantum efficiency (EQE) of the produced GaN nano-crystalline film were assessed. Responsivity (R_λ_) can be calculated using Eq. (4)^57,96,100,101^, which stands as a significant figure of merit. Both Eqs. (5) and (6)^102–105^ represent detectivity (D*) and external quantum efficiency (EQE), respectively

4 $$ {\text{R}}_{{\uplambda }} = \frac{{{\text{I}}_{{{\text{ph}}}} }}{{\text{P}}} $$

I_ph_ is the photocurrent (Ampere), and P is the incident power (Watt)^106,107^.

5 $$ {\text{D}}_{{\uplambda }}^{*} = \frac{{{\text{R}}_{{\uplambda }} {\text{A}}^{1/2} }}{{\sqrt {2{\text{qI}}_{{{\text{dark}}}} } }} $$

where A is the area of photodetector, ${\mathrm{I}}_{\mathrm{dark}}$ is the dark current of photodetector, and q is the electron charge^108–111^.

6 $$ {\text{EQE}} = \frac{{1240{\text{ R}}_{{\uplambda }} }}{{\lambda_{nm} }} $$

**Figure 14:**
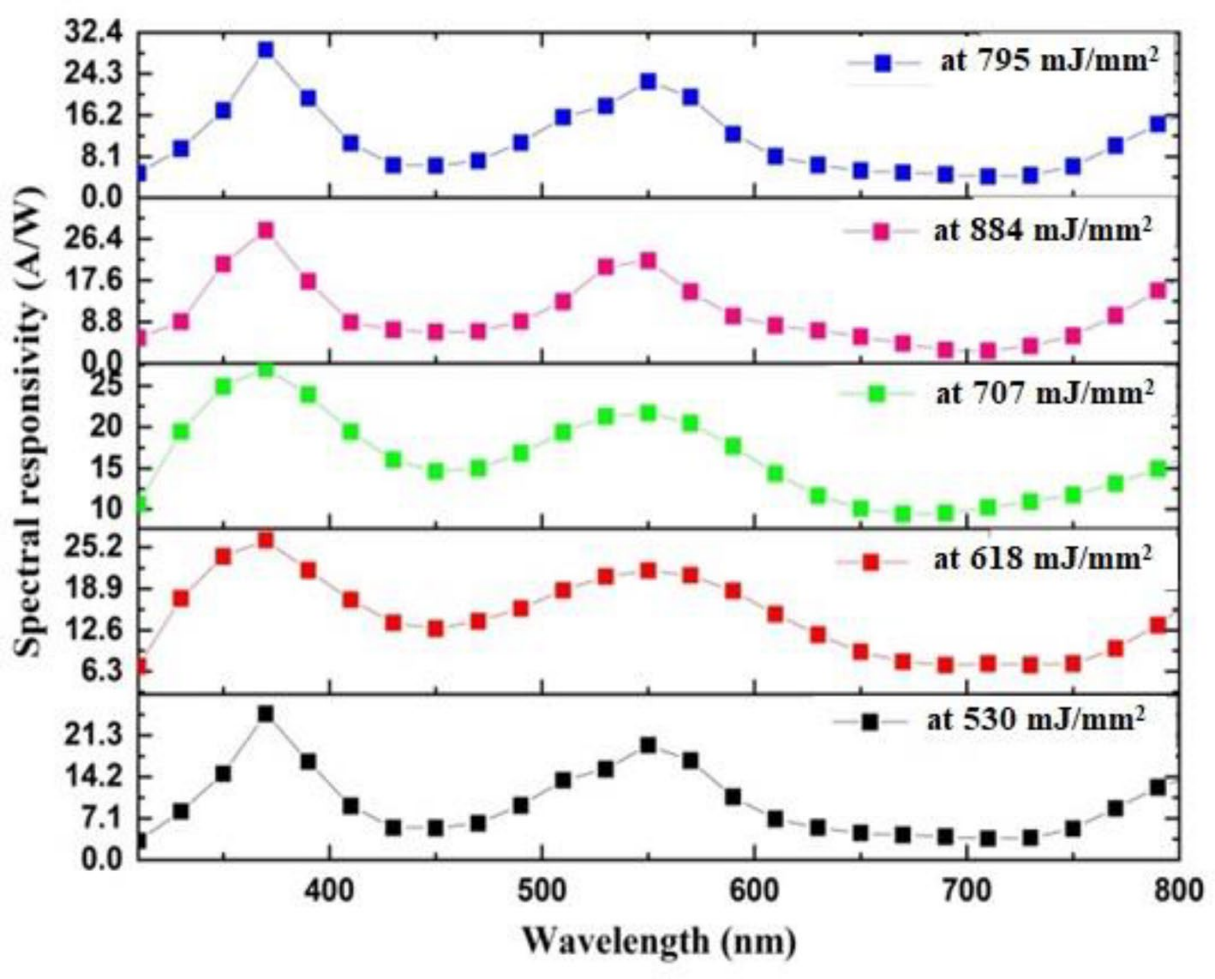
Spectral responsivityof fabricated GaN/P-Si nanostructure at 355 nm, 300 °C and different laser fluences.

Figure 14 depicts the responsivity of the structure when subjected to varying laser intensities operating between 350 and 850 nm. Three response peaks of 29.010 A/W at 370 nm and 22.761 A/W at 550 nm were observed in the fabricated GaN on P-Si nanostructure at 795 mJ/mm^2^, which can be attributed to the larger surface area, extended depletion layer width, and increased minority carrier diffusion length^112^.

**Figure 15:**
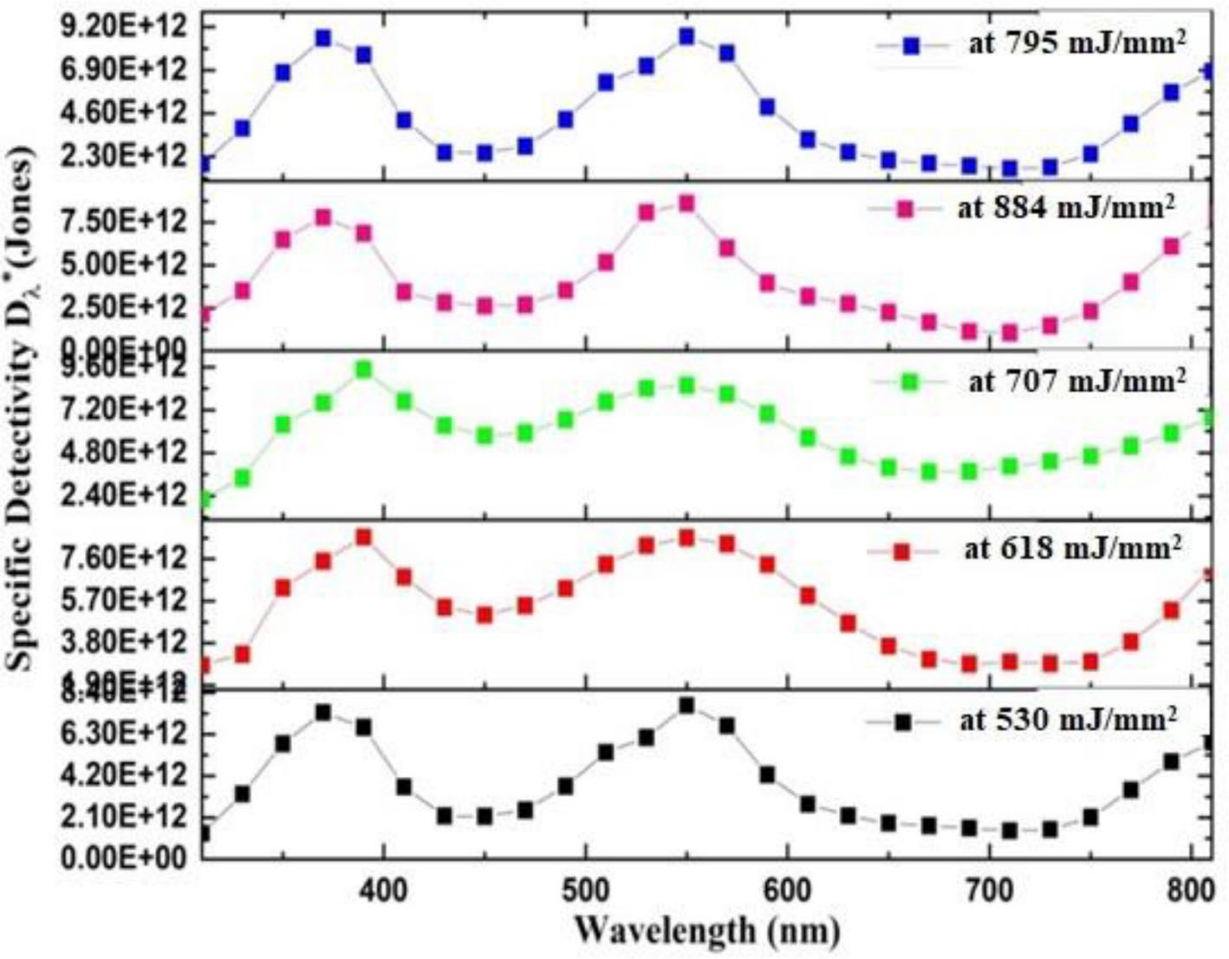
Detectivity of GaN/P-Si heterojunction photodetectors fabricated at different laser fluences under − 3 V bias.

Figure 15 depicts variation of detectivity (D*) with wavelength of the GaN/P-Si photodetectors. Two peaks were observed at 355 nm and 550 nm.

**Figure 16:**
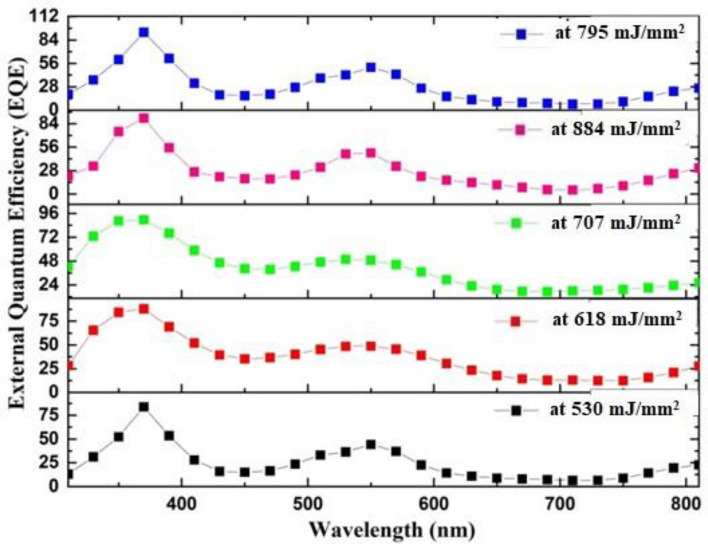
External quantum efficiency of GaN/P-Si nanostructure fabricated at different laser fluences.

Figure 16 depicts the EQE of GaN/PSi photodetectors fabricated at various laser fluences. Among these, the photodetector fabricated at 795 mJ/mm^2^ achieved the highest EQE values: 93.240% at 370 nm and 51.30% at 550 nm. The GaN/PSi heterojunction photodetector fabricated at 795 mJ/mm^2^ demonstrated a high EQE due to the direct relationship with Eq. (6), driven by its strong spectral response. Enhancing the reverse bias voltage can improve the collection efficiency of photogenerated carriers, allowing for the creation of a fully depleted photodetector^113,114^.

**Figure 17:**
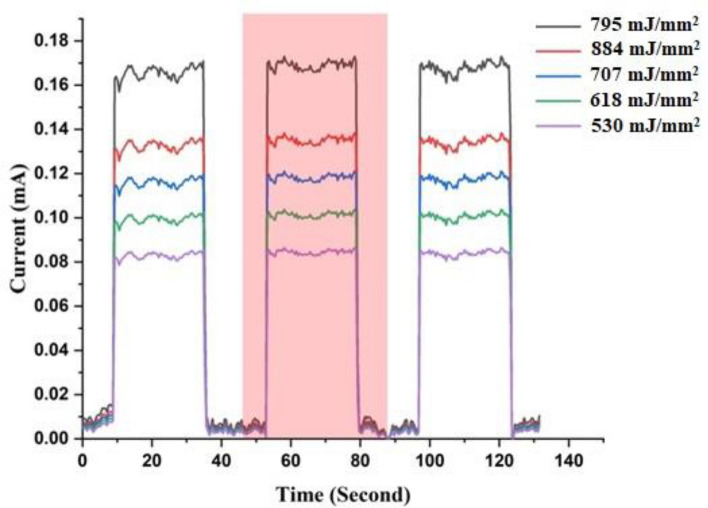
A switching characteristic of GaN/P-Si nanostructure fabricated at different laser fluences.

**Figure 18:**
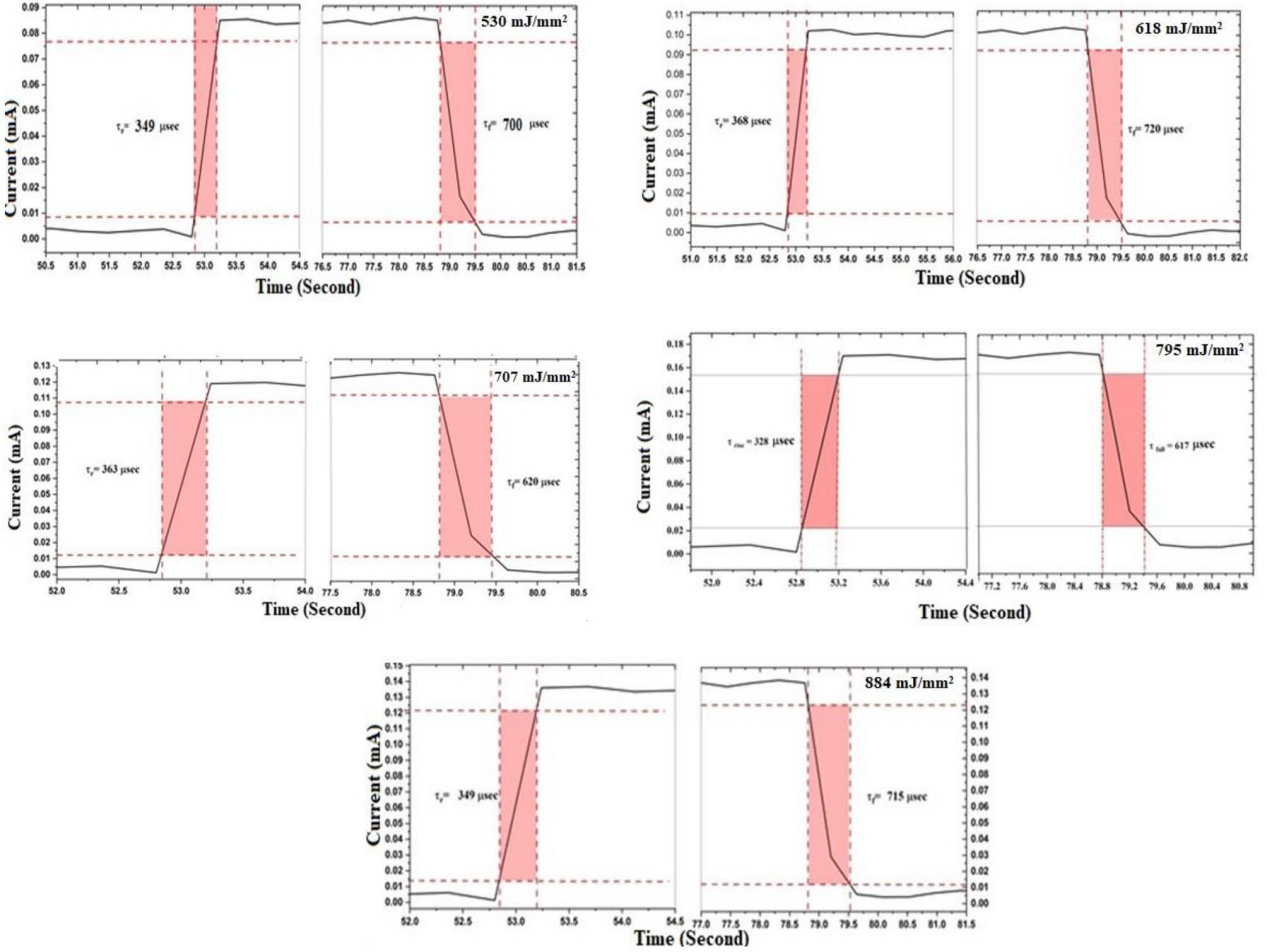
Rise time and fall time of GaN/PSi heterojunction photodiode fabricated at different laser fluences.

Figures 17 and 18 depict the dynamic photoresponse switching of the photodetectors deposited at various laser fluences. Three distinct switching cycles were conducted, each with an 18-s off period followed by a 25-s on period. Rise and fall times of the fabricated GaN/P-Si nanostructure were measured from 10 to 90% of the peak signal and from 90 to 10% of the peak signal, respectively. The photodetector prepared with a laser fluence of 795 mJ/mm^2^ exhibits a switching characteristic, with a measured rise time of 363 μs and a fall time of 711 μs.

The fabricated GaN/P-Si nanocrystalline film at 795 mJ/mm^2^ exhibited the best performance, with a responsivity of 29.010 A/W at 370 nm, a detectivity of 8.61 × 10^12^ Jones, and an external quantum efficiency of 93.240%. Additionally, it demonstrated a fast response rise time of 328 and a fall time of 617, outperforming Jiang et al. (2022), who fabricated a GaN/Si UV photodetector using a chemical vapor deposition process. Their device showed a responsivity of 71.4 mA/W, detectivity of 7.1 × 10^8^ Jones, external quantum efficiency of 24.3%, and a response time of 0.2/7.6 s^115^. Table 5 provides a summary of the figures of merit for the GaN/PSi photodetectors fabricated at various laser fluences.

**Table 5:** Shows the results of testing a manufactured GaN/P-Si nanostructure fabricated at different laser fluences.

Laser fluence (mJ/mm^2^)	Spectral responsivity (A/W)	Specific detectivity (Jones)	External quantum efficiency (%)	Rise time ($\mathrm{\mu s}$)	Fall time ($\mathrm{\mu s}$)
530	25.011 at 370 nm	7.40 × 10^+12^	83.801	349	700
	19.620 at 550 nm	7.750 × 10^+12^	44.212		
618	26.271 at 370 nm	7.51 × 10^+12^	88.051	368	720
	21.660 at 575 nm	8.51 × 10^+12^	48.812		
707	27.081 at 370 nm	7.60 × 10^+12^	89.512	363	620
	21.701 at 555 nm	8.50 × 10^+12^	48.911		
795	29.010 at 370 nm	8.61 × 10^+12^	93.240	328	617
	22.762 at 555 nm	8.70 × 10^+12^	51.306		
884	28.192 at 370 nm	7.81 × 10^+12^	90.702	349	715
	21.811 at 555 nm	8.60 × 10^+12^	49.110		

## Conclusion

Nanostructured GaN/PSi photodetectors were successfully fabricated using the pulsed laser deposition method at various laser fluences. The GaN nanostructure film deposited at 795 mJ/mm^2^ exhibited high crystalline peaks with a large crystallite size, indicating favorable structural characteristics. Spectroscopically, this film exhibited a shorter wavelength of 260 nm and a high energy gap of 4.76 eV. Morphologically, the film showed uniform, homogeneous spherical particles resembling cauliflower, with a thickness of 383.36 nm. Additionally, a uniform deposition yielded the largest average particle diameter (178.8 nm) and average surface roughness (50.61 nm).

Furthermore, performance characteristics were assessed for GaN nanostructures prepared using a laser fluence of 795 mJ/mm^2^. Due to the energy gap of GaN material, the responsivity under 3 V exhibited maximum values: responsivity of approximately 29.03 A/W, detectivity of 8.6 × 10^12^ Jones, and an external quantum efficiency of 97.2% at 370 nm. Similarly, at 575 nm, the responsivity measured around 19.86 A/W, detectivity of 8.9 × 10^12^ Jones, and an external quantum efficiency of 50.89%.

Additionally, three switching cycles with an 18-s off period and a 25-s on period were illuminated with a power of 100 mW/cm^2^. The rise time of the fabricated GaN/P-Si nanostructure was 328 μs, while the fall time was 617 μs. A strong correlation was observed between the optimum laser Fluence (795 mJ/mm^2^) and the achieved GaN nanostructure performance characteristics.

## Data Availability

Correspondence and requests for materials should be addressed to Makram A. Fakhri, Haneen D. Jabbar, and Evan T. Salim.
